# Mesenchymal stem cells elicits Anti-PD1 immunotherapy by targeted delivery of CX3CL1

**DOI:** 10.3389/fphar.2023.1136614

**Published:** 2023-02-08

**Authors:** Jize Liu, Xiaomin Ma, Chuxuan Liu, Yang Cheng, Bingjun Li, Wenjie Zhang, Runzhi Zeng, Qishuai Chen, Yun Zhang, Sanyuan Hu

**Affiliations:** ^1^ Department of General Surgery, The First Affiliated Hospital of Shandong First Medical University and Shandong Provincial Qianfoshan Hospital, Jinan, China; ^2^ Shandong First Medical University and Shandong Academy of Medical Sciences, Jinan, China; ^3^ Department of General Surgery, Shandong Provincial Qianfoshan Hospital, Cheeloo College of Medicine, Shandong University, Jinan, China

**Keywords:** colorectal cancer, mesenchymal stem cells, anti-PD1 antibodies, macrophages, tumor microenvironment, CD8^+^ TCELL, immunotherapy

## Abstract

Anti-PD1/PDL1 monotherapy has failed to acquire sufficiently ideal results in most solid tumors. Mesenchymal stem cells (MSCs) have been reported to exert therapeutic effects on some tumors, but the functions of MSCs in colorectal cancer (CRC) need further research. In this study, we aimed to investigate the therapeutic effect and the improvement of sensitivity of MSCs to anti-PD1 antibodies (αPD1) in CRC and to evaluate the possible mechanism. The relative distribution of immune cells in tumor microenvironment was examined after the mice were treated with MSC and/or αPD1. Our study revealed that MSC recruits CX3CR1^high^ macrophages and promotes M1 polarization to inhibit tumor growth *via* highly secretion of CX3CL1.The combination of MSC and αPD1 was superior to monotherapy against CRC. MSC inhibits PD1 expression on CD8^+^ T cells by facilitating M1 macrophage polarization, which promotes the proliferation of CD8^+^ T cells, thus improving the sensitivity to αPD1 therapy in CRC. Additionally, the above therapeutic effect disappeared after inhibiting the secretion of CX3CL1 in MSC. Our MSC-based immunotherapeutic strategy simultaneously recruited and activated immune effector cells at the tumor site, suggesting that the combination of MSC and αPD1 could be a potential therapy for CRC.

## 1 Introduction

Colorectal cancer (CRC) is a highly prevalent type of malignancy (Sung et al., 2021). Obesity is a risk factor for the initiation and progression of colorectal cancer, and causes glucose metabolism, lipid metabolism, and immune system disorders (Andersen et al., 2016; Bray et al., 2018). Accumulating evidence has shown that the increase in total cholesterol, unsaturated fatty acid levels, and chronic inflammatory status accompanied by obesity increases the risk of CRC (Song et al., 2015; Bull et al., 2020). With lifestyle changes and improvements in living standards, the number of patients with colorectal cancer will continue to increase (Clinton et al., 2020). Therefore, seeking more effective treatment strategies is becoming highly urgent. Immunotherapy has emerged as a treatment method in recent years. Unlike conventional treatment modalities, which directly attack tumors, immunotherapy aims to activate immune cells to kill tumor cells. Macrophages are predominant in the innate immune system and can activate CD8^+^ T cells to specifically attack tumor cells in the tumor microenvironment (TME) (Yin et al., 2021), which is recognized as the most effective immune response against tumors (Yang et al., 2021).

The programmed cell death protein-1 (PD-1) immune checkpoint is a critical traget in antitumor immunity. TME can inhibit the function of CD8^+^ T cells, attenuate their tumor-killing activity, and induce rapid exhaustion through PD1/PDL1 interaction (Ai et al., 2020). Therefore, blocking the PD1/PDL1 axis using monoclonal antibodies has been widely used in clinical treatment. However, in most solid tumors, a single anti-PD1/PDL1 antibodies therapy fails to acquire sufficiently ideal results (Ai et al., 2020). Reducing CD8^+^ T cells depletion, and thus effectively recruiting and activating more CD8^+^ T cells to kill tumors, will become a valid strategy to improve immunotherapy efficacy.

Macrophages, the largest immune cell population in the tumor microenvironment (TME), play a connection role in specifically recognizing tumor antigens and targeting activated CD8^+^ T cells (Klug et al., 2013). Based on the expression of surface molecules, mouse macrophages can be divided into two main subsets: F4/80^+^ CX3CR1^high^ macrophages or F4/80^+^ CX3CR1^low^ macrophages (Ziegler-Heitbrock et al., 2010). Presently, there are different opinions on the function of the two types of macrophages: in melanoma models, CX3CR1^high^ macrophages prevent melanoma metastasis by recruiting NK cells (Hanna et al., 2015); in lung carcinoma models, CX3CR1^high^ macrophages promote cancer progression by improving angiogenesis (Coffelt et al., 2010); in breast cancer models, CX3CR1^low^ macrophages are recruited to metastatic sites to promote cancer cell extravasation and subsequent growth (Qian et al., 2011). The functions of each macrophage subsets in CRC remain unclear. Additionally, these two subsets of macrophages can polarize in different or diametrically opposite directions when stimulated in different microenvironments (Rahman et al., 2017). Macrophages can polarize into M1 or M2 subtypes, which demonstrates their plasticity (Biswas and Mantovani, 2010). M1 macrophages are mainly characterized by obtaining antigen presentation function, effectively promoting T cell activation, preventing their depletion, and exerting antitumor immune effects against tumor cells (Mills and Ley, 2014). M2 macrophages facilitates tumor growth and progression by producing molecules that promote angiogenesis, tumor cell survival, and metastasis (Pollard, 2004; Condeelis and Pollard, 2006). Most types of TME can induce macrophages polarization into a M2-like tumor-promoting population; however, reversing the TME by changing macrophages polarization direction can effectively inhibit tumor growth and progression. Consequently, based on the high plasticity of macrophages, reversing the proportion of different macrophage subsets, and then recruiting and activating CD8^+^ T cells to achieve better tumor killing in the tumor microenvironment will be a feasible and suitable cancer immunotherapeutic approach.

Based on the literature, we identified an immunomodulatory tool, mesenchymal stem cells (MSC). MSC belongs to the autologous normal cell population and has a rich chemokine expression profile to recruit and modify various immune cells (Ahmadian Kia et al., 2011), which affects tissue metabolism and inflammation and plays an important role in immunometabolism in tumors (Spallanzani, 2021). MSCs were divided into three categories according to their cell origin: bone marrow-derived MSCs (BM-MSCs), umbilical cord blood-derived MSCs (UCB-MSCs), and adipose tissue-derived MSCs (AT-MSCs) (Kern et al., 2006). AT-MSCs, as an ideal cells, have the following advantages: easy access, long-term *in vitro* culture, higher levels of expressed chemokine receptors, spontaneous recruitment to inflammation and tumorigenesis sites, minimal risk to donors, and no ethical issues (Kern et al., 2006). MSC can be used as an ideal “hand” to reshape macrophages in the TME and differentiate toward inhibition of tumors based on their advantages in immune regulation, tumor progression inhibition, avoidance of autoimmune rejection, and easy access (Aggarwal and Pittenger, 2005).

Therefore, this study aimed to explore the therapeutic effect of MSC in CRC and the feasibility of MSC to enhance the sensitivity of CD8^+^ T-αPD1 immunotherapy therapy by recruiting and engineering macrophages through abundant chemokine expression profiles.

## 2 Materials and methods

### 2.1 Cell lines and animals

MC38 cells were purchased from the American Type Culture Collection (ATCC), cultured in RPMI 1640 medium supplemented with 10% fetal bovine serum (FBS) and 1% penicillin/streptomycin in 5% CO_2_ at 37°C. The cells were passaged when they reached 80–90% confluence. Our experiments were performed using C57BL/6 mice (male, 6–8 w) and BALB/c nude mice (male, 6–8 w) weighing 18–22 g. C57BL/6 mice and BALB/c nude mice, purchased from Vital River Laboratory Animal Technology (Beijing, China), were housed in the animal laboratory of First Affiliated Hospital of Shandong First Medical University. All animal experimental procedures were approved by the First Affiliated Hospital of Shandong First Medical University.

### 2.2 Isolation, culture, and identification of MSCs from mouse adipose tissue

AT-MSCs were isolated from mouse subcutaneous adipose tissue using adipose tissue dissociation kit (Miltenyi Biotec) and plated in Dulbecco’s modified Eagle medium (DMEM) supplemented with 10% FBS and 1% penicillin/streptomycin. The following experiments used AT-MSCs between passages three and five. The phenotypic profile of MSCs was evaluated to confirm the identity of cells by flow cytometry analysis using anti-mouse antibodies against CD29, CD44, CD45, and CD34. The antibodies used for flow cytometry were purchased from Biolegend. These antibodies were diluted according to the manufacturer’s instructions (1:1,000).

### 2.3 Isolation and differentiation of murine BM-derived macrophages

Bone marrow-derived macrophages (BMDMs) were isolated from the femurs and tibias of six- to 8-week-old C57BL/6 mice. Cell debris was removed by passaging the suspension through a 100 μm nylon sterile strainer. Following washing with PBS for three times, 1 × 10^6^ cells were seeded on 12-well plates (Corning Costar). Cells were cultured in DMEM with 100 ng/mL M-CSF for 6 days in a cell incubator at 37°C and 5% CO_2_. Following these initial differentiation steps, cells were washed with PBS and incubated with complete medium or conditioned medium (CM) of MSC, MSC^*CX3CL1-*^, MSC co-cultured with MC38, and MSC ^*CX3CL1-*^ co-cultured with MC38 for two additional days.

### 2.4 *In vivo* tumor transplantation

Mice of the appropriate age were randomly divided into four groups: cIg-treated group (as the control group), αPD1-treated group, MSC × cIg-treated group, and αPD1 × MSC mixed treatment group. Orthotopic cancer models were established by subcutaneous tumor implantation in the flank. Tumor transplantation were performed in an anesthetized mouse. Tumor cells (5 × 10^5^) were injected alone or co-injected with mesenchymal stem cells (1 × 10^4^) s.c. In a volume of 100 μm. The immune checkpoint-blocking antibody anti-PD-1 and control antibody IgG (cIg) was purchased from Bio X Cell. αPD1 and cIg (200 ug/mouse, 2 mg/ml) were administered intraperitoneally (i.p.), once every 3 days.The tumor volume of the mice was monitored and administered every 3 days from day 4. The tumor volume was measured using a caliper and calculated as (length × width^2^)/2. For the resection experiment, primary tumors (2 cm in the largest diameter) were resected on day 16 after subcutaneous injection under general anesthesia and were used for subsequent experiments. The process of knockdown group in subsequent experiments was the same as normal group.

### 2.5 *In vivo* depletion experiments

Clodronate Liposomes (CL) were purchased from Liposomas. Mice of the appropriate age were randomly divided into two groups:MSC × PBS group, MSC × CL group. Macrophages depletion started 6 days after tumor challenge with CL i.p. Twice a week at a dosage of 0.05 mg/g body weight. As a control, PBS liposomes were used in the experiments.

### 2.6 CCK8 assay

MC38 group: Tumor cells were incubated with complete medium; MC38 + MSC group:Tumor cells were incubated CM of MSC. Then tumor cell proliferation was assessed using the cell counting Kit-8 (CCK-8) (Dojindo, Japan) on days 1 and 2 according to the manufacturer’s instructions. The cells were seeded in 96-well microplates (Corning, United States) at a density of 1 × 10^4^ cells/well in 100 μL of medium for culture. The absorbance was measured at 450 nm using a microplate reader (BioTek). All experiments were performed at least three times.

### 2.7 EdU staining assay

MC38 group: Tumor cells were incubated with complete medium; MC38 + MSC group:Tumor cells were incubated CM of MSC. EdU staining was used to analyze cancer cell proliferation according to the manufacturer’s instructions (Beyotime). Cells were fixed in 4% paraformaldehyde for 20 min at room temperature.

### 2.8 T-cell proliferation assay

CD8^+^ T cells were isolated from mouse spleen tissue using the CD8^+^ T Cell Isolation Kit (Miltenyi Biotec) according to the manufacturer’s instructions. T-cell blasts (10^6^/mL in PBS) were labeled with 5 mM CFSE (BD Biosciences) for 10 min at 37°C. Staining was terminated by the addition of fetal calf serum. After two washes with the medium, T cells were incubated with BMDM, MSC, MSC ^*CX3CL1-*^, MSC co-cultured with BMDM, and MSC ^*CX3CL1-*^ co-cultured with BMDM. The medium used for the above groups was the CM of MC38 cells. Cell division, as evidenced by the reduction in fluorescence intensity by half, was analyzed by flow cytometry.

### 2.9 Immunofluorescence staining

The tissues were fixed in formalin for 24 h, embedded in paraffin, and sectioned. De-paraffinized tissue sections were serially incubated in xylene and 100%, 90%, 70% ethanol, and water. Antigen retrieval was performed using a citrate antigen retrieval solution in a microwave. Subsequently, the sections were blocked with 30% goat serum for 1 h at room temperature and incubated with primary antibodies overnight at 4°C. The sections were then washed twice with washing buffer and incubated with a secondary antibody.

### 2.10 Flow cytometry

To analyze tumor-infiltrating immune cells, subcutaneously tumors were dissected and transferred into RPMI 1640 medium, disrupted mechanically with scissors, digested for 1 h at 200 rpm/min using a mouse tumor dissociation kit at 37°C, and dispersed through a 100 μm cell strainer to remove residual tissue (BD Biosciences). Single cells were washed and stained with antibodies for 30 min at room temperature. Dead cells were excluded by staining with the Zombie fixable viability kit for 30 min (BioLegend). Fluorescence data were acquired on a Sony ID7000 (Sony) and analyzed using FlowJo software. Tumor-infiltrating cells isolated from tumor tissues, T cells derived from the spleen, or BMDMs were processed for surface labeling with antibodies against CD45, CD11b, F4/80, CX3CR1, CD3, CD8, PD1, TIGIT, TIM3, CD206, and I^A^I^E^. The antibodies used for flow cytometry were purchased from Biolegend. These antibodies were diluted according to the manufacturer’s instructions (1:1,000).

### 2.11 RT-qPCR

mRNA was isolated using the RNeasy Mini Kit (Qiagen, Hilden, Germany) according to the manufacturer’s instructions. cDNA was synthesized using a PrimeScript RT Reagent Kit (Takara). qPCR was performed using the SYBR Green PCR Master Mix Kit (Takara). β-actin was used for mRNA normalisation.mRNA expression levels of target genes were calculated using the DDCt method. The primers were designed as follows: 5′- CAT​TGC​TGA​CAG​GAT​GCA​GAA​GG -3′ and 5′- TGC​TGG​AAG​GTG​GAC​AGT​GAG​G -3′ for beta-actin. 5′- ACG​AAA​TGC​GAA​ATC​ATG​TGC -3′ and 5′- CTG​TGT​CGT​CTC​CAG​GAC​AA -3′ for CX3CL1.

### 2.12 ELISA

Cells were cultured in 24-well plates for 2 days. The supernatants were collected and stored in a refrigerator at −80°C until measurement. CX3CL1 secreted into the supernatant was quantified using a mouse CX3CL1 ELISA kit (Abcam) according to the manufacturer’s instructions.

### 2.13 Adenovirus transduction in MSCs

MSCs were infected with adenovirus at a multiplicity of infection (MOI) of 500. MSCs were plated on 6-well plates (1 × 10^5^/well). On the next day, cells were washed twice with PBS and transduced with adenovirus for 36 h. All adenoviruses contained mCherry, and the transduction efficiency in MSCs was validated by mCherry expression under a microscope before subsequent experiments. Successful knockdown of CX3CL1 in MSCs was validated by RT-qPCR and ELISA.

### 2.14 Statistical analysis

All results are expressed as the mean ± SD. Differences were assessed using Student’s t-test or two-way ANOVA (when the means of more than two groups were compared) followed by a Bonferroni multiple comparison test. Data analyses were performed using GraphPad Prism (GraphPad Software, San Diego, California United States of America). A *p*-value < 0.05 was considered statistically significant.

## 3 Results

### 3.1 MSC inhibits the growth and proliferation of colorectal cancer and enhances sensitivity to αPD1

**FIGURE 1:**
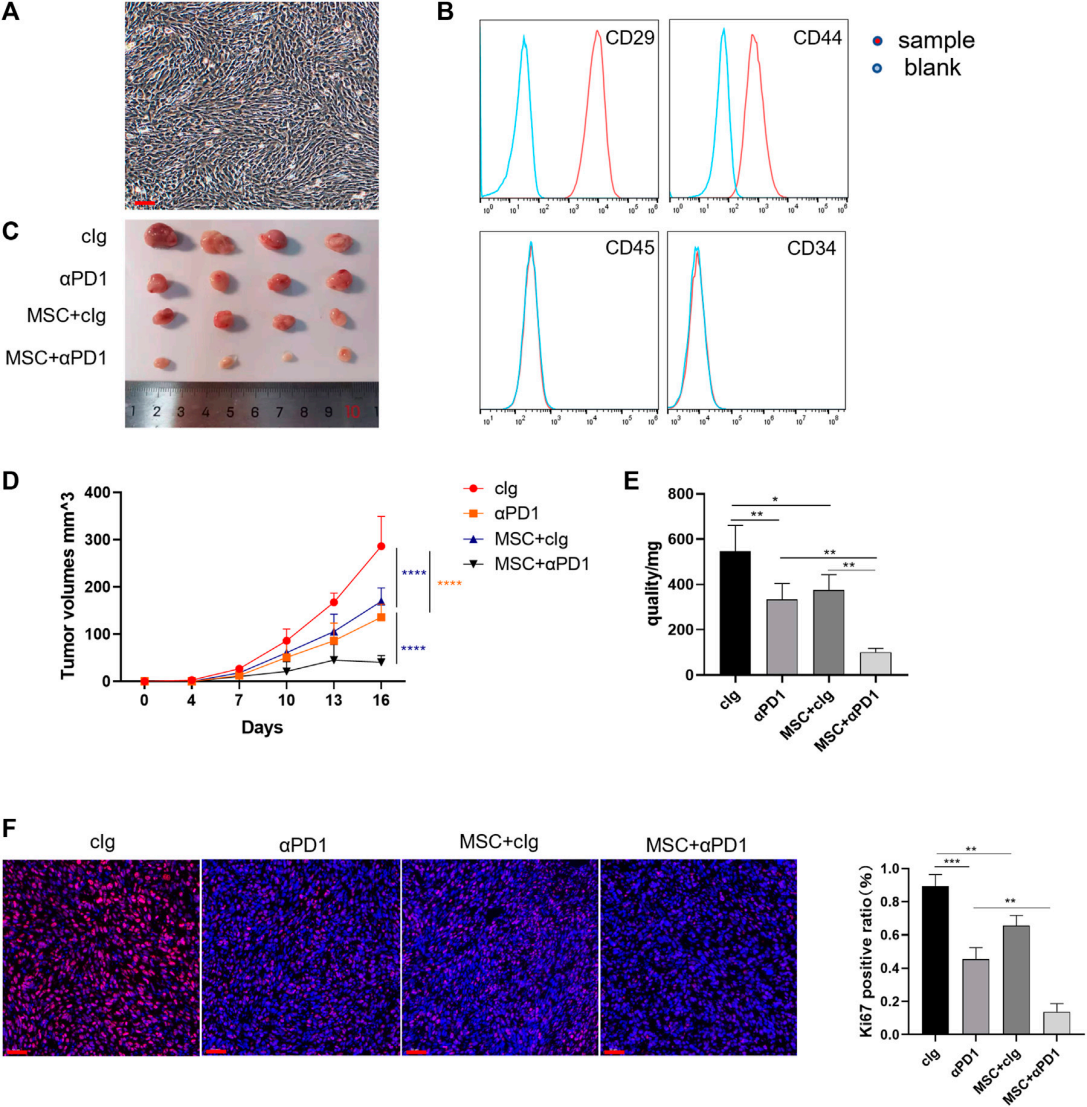
The therapeutic effect of MSC in CRC **(A)** Brightfield image showing morphology of mouse MSCs **(B)** Characterization of MSCs isolated from C57BL/6 mice analysed by FACS **(C)** Tumor image of C57BL/6 mice in the flank (day 16), after injection of MC38 alone or co-injected with MSCs [cIg-treated group, *n* = 4; αPD1-treated group, *n* = 4; MSC×cIg-treated group, *n* = 4; αPD1 × MSC mixed treatment group *n* = 4. ] **(D–E)** The tumor volume and weight of mice were compared in each group **(F)** The tumor tissues removed from C57BL/6 mice were analyzed using immunofluorescence staining for expression of Ki-67 (scale bars, 50 μm). Significance identification: ns, *p* ≥ 0.05; *, *p* < 0.05; **, *p* < 0.01; ***, *p* < 0.001. Control group:cIg-treated group; MSC, MSC-treated group.

First, we isolated and characterized AT-MSCs from the mice. Microscopically, the mesenchymal stem cells were spindle-shaped and grew spirally adherent at the bottom of the dish (Figure 1A), which was consistent with the morphological characteristics of AT-MSCs. Then these cells were identified by flow cytometry and we found that they expressed MSC-specific markers, such as CD29 and CD44, but lacked leukocyte marker CD45 and hematopoietic or endothelial progenitor cell marker CD34 (Figure 1B), indicating the high purity of the AT-MSCs. To investigate whether MSC could regulate CRC growth and enhance the efficacy of αPD1, the following groups were designed: control group, αPD1-treated group, MSC × cIg-treated, and αPD1 × MSC mixed treatment. We found that comparded with control group, both αPD1 and MSC inhibited the growth of CRC, and the inhibitory effect was more apparent in the mixed treatment group (Figure 1C). This result was also supported by the volume and weight of the tumors (Figures 1D, E). Ki67 immunofluorescent staining was performed in tumor tissues, and it was found that the positive ratio of Ki67 was attenuated in both the αPD1-treated and MSC × cIg-treated groups compared with the control group, and significantly decreased in the mixed treatment group (Figure 1F). The above results showed that MSC, as an immunotherapeutic tool, inhibited the growth and proliferation of CRC and significantly enhanced the sensitivity of CRC to αPD1 treatment.

### 3.2 MSC has no direct suppressive effect on colorectal cancer growth and proliferation

**FIGURE 2:**
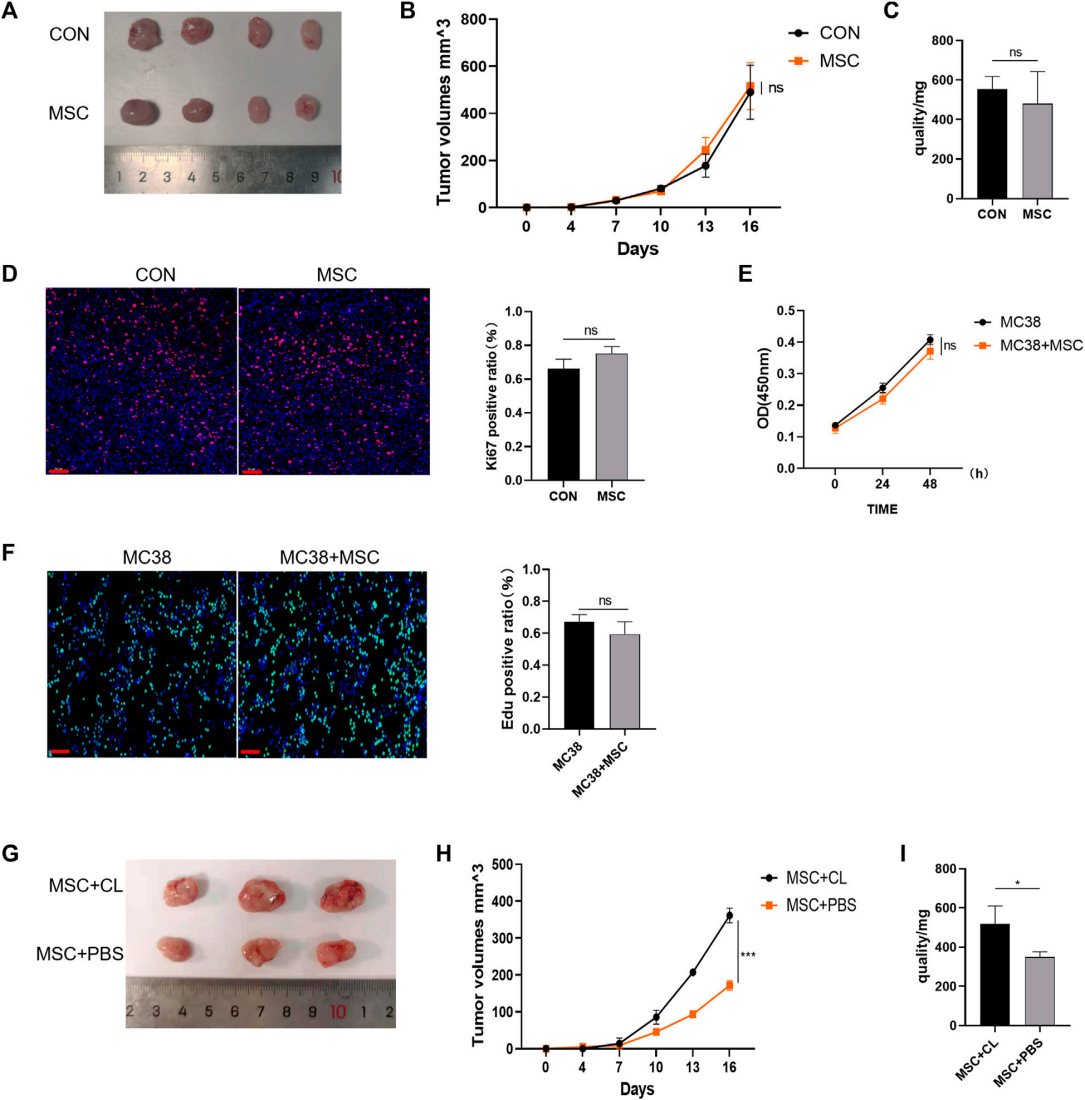
MSC has no direct suppressive function in CRC **(A)** Tumor image of BALB/c nude mice in the flank (day 16), after injection of MC38 + PBS (control group) or MC38 + MSC (MSC-treated group) **(B–C)** The tumor volume and weight of mice were compared with the control group (control group, *n* = 4; MSC-treated group, *n* = 4.). **(D)** Ki-67 immunofluorescence on CRC tissues of mice (scale bars, 50 μm) **(E)** CCK8 assays analysis **(F)** EdU incorporation was analyzed by immunofluorescence on CRC tissues of mice (scale bars, 50 μm) **(G)** Tumor image of C57BL/6 mice. Macrophages were removed by Clodronate Liposomes. As a control, PBS liposomes were used in the experiments (*n* = 3) **(H–I)** The tumor volume and weight of C57BL/6 mice (*n* = 3). Significance identification: ns, *p* ≥ 0.05; *, *p* < 0.05; **, *p* < 0.01; ***, *p* < 0.001. CON, control group; MSC, MSC-treated group.

Next, the mechanism of action of MSC were explored. BALB/c nude mice (immunocompromised mice) were subcutaneously injected with MC38 alone or co-injected with MSCs into the control or MSC-treated groups. Our results showed no significant difference in tumor size between the MSC-treated and control groups (Figure 2A). The tumor growth curve and weight also showed that there was no significant difference in these groups. (Figures 2B,C). Tumor tissues were taken for Ki67 immunofluorescent staining, with the Ki67 positive ratio indicating no significant difference in the proliferation ability between the two groups (Figure 2D). Then, *in vitro* experiments were performed to validate these results. CRC cells were treated with MSC culture supernatant, and CCK-8 assay was performed 24h and 48 h later to detect cell proliferation. There was no significant difference in proliferation between the MSC culture supernatant treatment group and the control group at the two-time points (Figure 2E). EdU staining also showed no significant difference in proliferation ability between the two groups (Figure 2F).

Therefore, MSC had no direct inhibitory effect on the growth and proliferation of CRC cells. As BALB/c nude mice are immunodeficient, we speculated that MSC might play an inhibitory role in immune cells. TME is infiltrated by multiple types of immune cells, and as the largest immune cell population, macrophages play an crucial role during cancer progression. We designed an experiment to deplete macrophages using Clodronate Liposomes from C57BL/6 mice and found that the inhibitory effect of MSC on CRC growth disappeared compared with the MSC group, according to the tumor size, volume, and mass comparison (Figures 2G–I). Thus, we speculate that macrophages may mediate the inhibitory effect on CRC growth and proliferation.

### 3.3 MSC increases recruitment of the CX3CR1^high^ macrophages and facilitates M1 polarization

**FIGURE 3:**
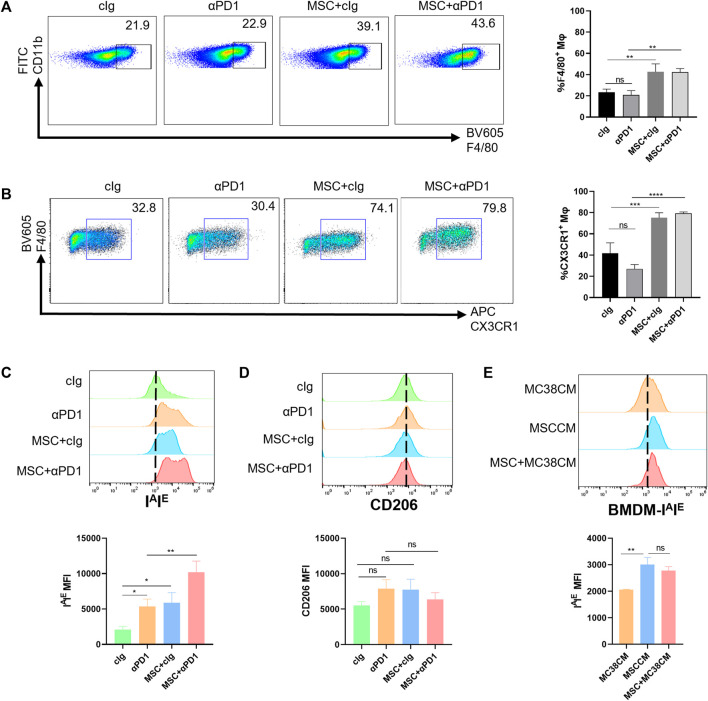
The recruitment and modification effect of MSC on macrophages **(A)** Tumor-infiltrating CD11 b^+^ F4/80^+^ macrophages were analyzed by FACS 16 days after the injection of MC38 alone or co-injected with MSCs in C57BL/6 mice (gate on CD11 b^+^) (*n* = 3) **(B)** Tumor-infiltrating F4/80^+^ CX3CR1 ^high^ macrophages were analyzed by FACS(gate on F4/80^+^) (*n* = 3) **(C–D)** Expression of I^A^I^E^ and CD206 in CX3CR1 ^high^ macrophages were analyzed by FACS (*n* = 3) **(E)** Expression of I^A^I^E^ in CM stimulated BMDMs were analyzed by FACS (*n* = 3). Significance identification: ns, *p* ≥ 0.05; *, *p* < 0.05; **, *p* < 0.01; ***, *p* < 0.001. Mφ:macrophage.

Based on previous inferences, we evaluated the effects of MSC on the macrophages. Fresh tumor tissues were digested into single-cell suspensions and analyzed by flow cytometry (gating strategies in Supplementary Figure S1). We found that MSC increased the proportion of macrophages infiltrating the TME (Figure 3A). Further division of the macrophage population revealed that the increased macrophages were dominated by the CX3CR1 ^high^ macrophages subset (Figure 3B). In mice, M1 macrophage polarization is characterized by increased expression of I^A^I^E^ and M2 macrophage polarization by increased expression of CD206. So we analyzed the polarization of CX3CR1 ^high^ macrophages. We found that both αPD1 and MSC promoted CX3CR1 ^high^ macrophages M1 polarization, which was significantly stimulated by αPD1 × MSC mixed treatment (Figure 3C). We also analyzed M2 polarization in CX3CR1 ^high^ macrophages, and various treatments had no significant effect on M2 polarization in this group of cells (Figure 3D). Additionally, mouse BMDM were extracted and stimulated with CM of MC38, MSC, or MSC co-cultured with MC38. We found that CM of MSC or MSC co-cultured with MC38 could promote M1 macrophage polarization by paracrine signaling (Figure 3E), further validating our results from *in vitro* experiments. Thus, we found that MSC can increase recruitment of the CX3CR1^high^ macrophages and facilitates M1 polarization of this subset.

### 3.4 CX3CL1-knockdown abrogated MSC inhibition of colorectal cancer, improvement of sensitivity to αPD1 treatment, and modification of CX3CR1^high^ macrophage

**FIGURE 4:**
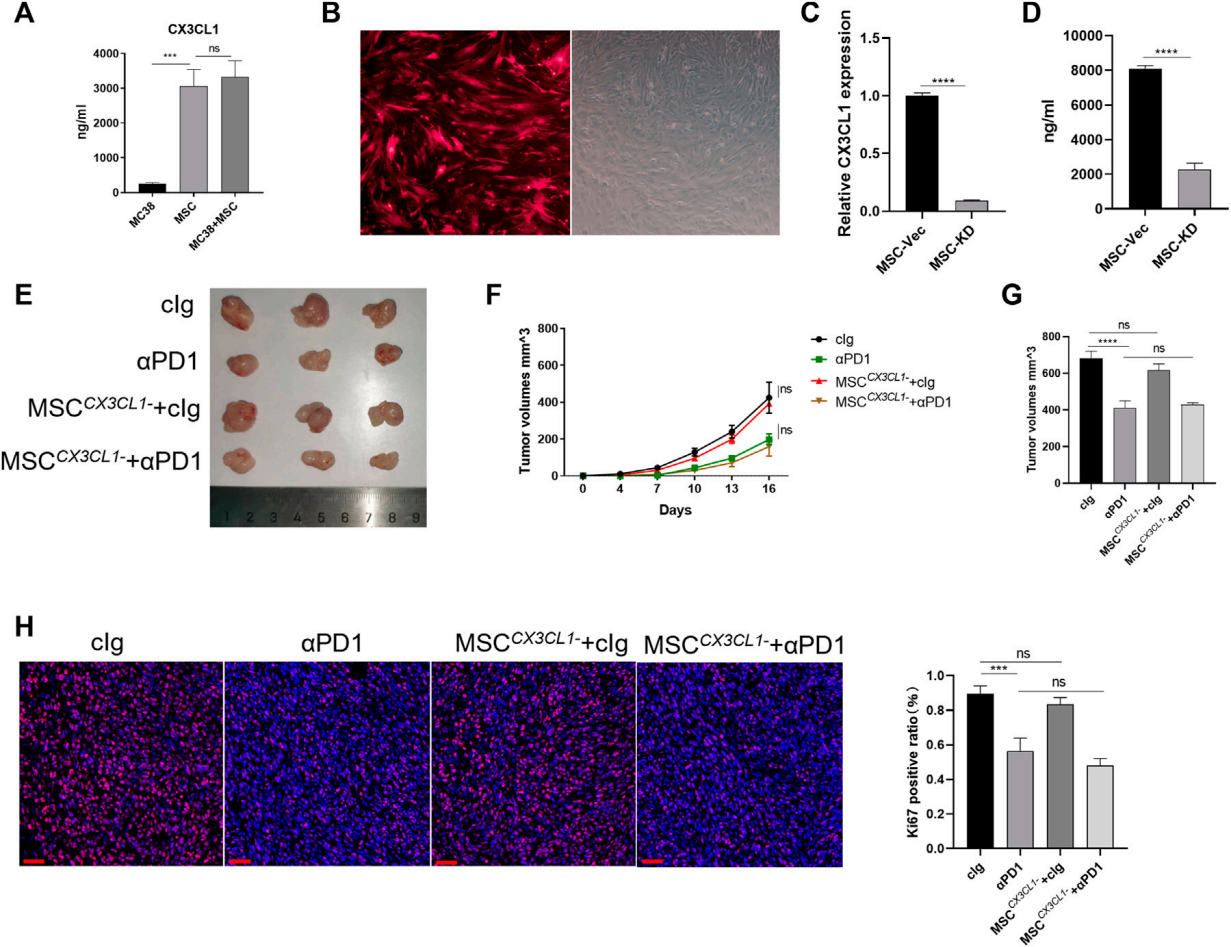
CX3CL1 knockdown abrogated MSC suppression of CRC **(A)** ELISA assay for CX3CL1 level in the supernatant of MC38, MSC and MC38 co-culture with MSC **(B)** Fluorescent microscope images following transfection of MSC with adenovirus **(C–D)** Expression of CX3CL1 in MSC^*CX3CL1-*^ was analyzed using RT-qPCR and ELISA **(E)** Tumor image of C57BL/6 mice in the flank (day 16), after injection of MC38 alone or co-injected with MSC ^*CX3CL1-*^ [cIg-treated group (control group), *n* = 3; αPD1-treated group, *n* = 3; MSC ^*CX3CL1-*^-treated group, *n* = 3; αPD1 × MSC ^*CX3CL1-*^ mixed treatment group *n* = 3.] **(F–G)** The tumor volume and weight of mice were compared in each group **(H)** Ki-67 immunofluorescence on CRC tissues of mice (scale bars, 50 μm). Significance identification: ns, *p* ≥ 0.05; *, *p* < 0.05; **, *p* < 0.01; ***, *p* < 0.001.

As a ligand for CX3CR1, CX3CL1 play an important role in recruiting and modifing specific immune cells *via* CX3CL1/CX3CR1 chemokine pathway (Helmke et al., 2019). CX3CL1 secretion in MC38 cells and MSC were examined. We found that MC38 cells barely secreted CX3CL1, while MSC secreted CX3CL1 (Figure 4A). We speculate that CX3CL1 hypersecretion by MSC may be the main reason for the recruitment and engineering of CX3CR1^high^ macrophages. Thus, we constructed adenoviruses with knocked-down CX3CL1 and transfected MSC with them, which showed successful transfection by fluorescence microscopy (Figure 4B). We then validated the knockdown efficiency at the gene *versus* protein level, and the results showed that the CX3CL1 knockdown cell model was successfully constructed (Figure 4C). To observe the effect of MSC on CRC proliferation and sensitivity to αPD1 treatment after the knockdown of CX3CL1, we performed subcutaneous tumor analysis again. The results showed no significant difference in tumor size, volume, and mass between the MSC ^*CX3CL1-*^cIg-treated group and the control group. Additionally, there was no significant difference in tumor size, volume, and mass between the αPD1 × MSC ^*CX3CL1-*^ mixed treatment group and the αPD1-treated group (Figures 4C–E). Ki67 immunofluorescence staining was performed in tumor tissues, and the results showed no significant difference in the Ki67 positive rate between the MSC ^*CX3CL1-*^ and control groups. Furthermore, there was no significant difference in the Ki67 positive rate between the αPD1 × MSC ^*CX3CL1-*^ mixed and αPD1 treatment groups (Figure 4H). Based on the above results, we speculated that MSC inhibited the growth and proliferation of CRC by secreting CX3CL1 and improved the αPD1 treatment sensitivity of CRC. These effects disappeared after the knockdown of MSC CX3CL1 secretion.

**FIGURE 5:**
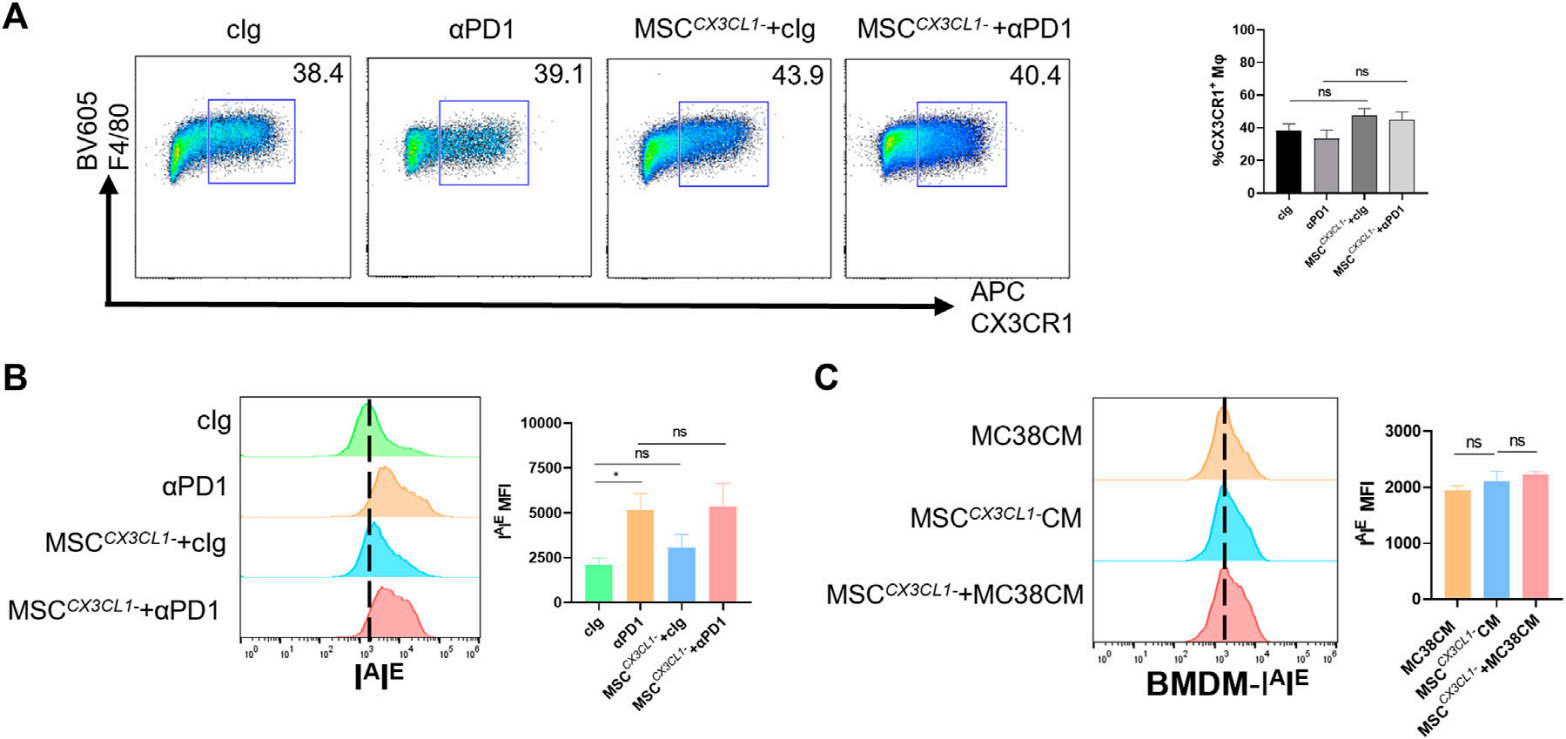
CX3CL1 knockdown abrogated MSC effect for macrophages **(A)** Tumor-infiltrating F4/80^+^ CX3CR1 ^high^ macrophages were analyzed by FACS (gate on F4/80^+^) (*n* = 3) **(B)** Expression of I^A^I^E^ in CX3CR1 ^high^ macrophages was analyzed by FACS (*n* = 3) **(C)** Expression of I^A^I^E^ in CM stimulated BMDMs was analyzed by FACS (*n* = 3). Significance identification: ns, *p* ≥ 0.05; *, *p* < 0.05; **, *p* < 0.01; ***, *p* < 0.001. Mφ:macrophage.

Next, the above samples were analyzed by flow cytometry, and results showed that there were no significant differences in the percentage of CX3CR1^high^ macrophages in TME between the MSC ^*CX3CL1-*^-treated group compared with the control group or the αPD1 × MSC ^*CX3CL1-*^ mixed treatment group compared with the αPD1-treated group (Figure 5A). Further analysis of the polarization status of CX3CR1^high^ macrophages, the MSC ^*CX3CL1-*^- treated group compared with the control group, and the αPD1 × MSC ^*CX3CL1-*^ mixed treatment group compared with the αPD1-treated group revealed that the effect of MSC on promoting CX3CR1^high^ macrophages M1 polarization disappeared after knockdown of CX3CL1 (Figure 5B). *In vitro* macrophage co-culture experiments confirmed this conclusion (Figure 5C). Thus, we found that CX3CL1-knockdown abrogated MSC inhibition of colorectal cancer, improvement of sensitivity to αPD1 treatment, and modification of CX3CR1^high^ macrophages.

### 3.5 MSC promotes the proliferation of CD8^+^ T cells and decreased PD1 expression in CD8^+^ T cell by engineering macrophages

**FIGURE 6:**
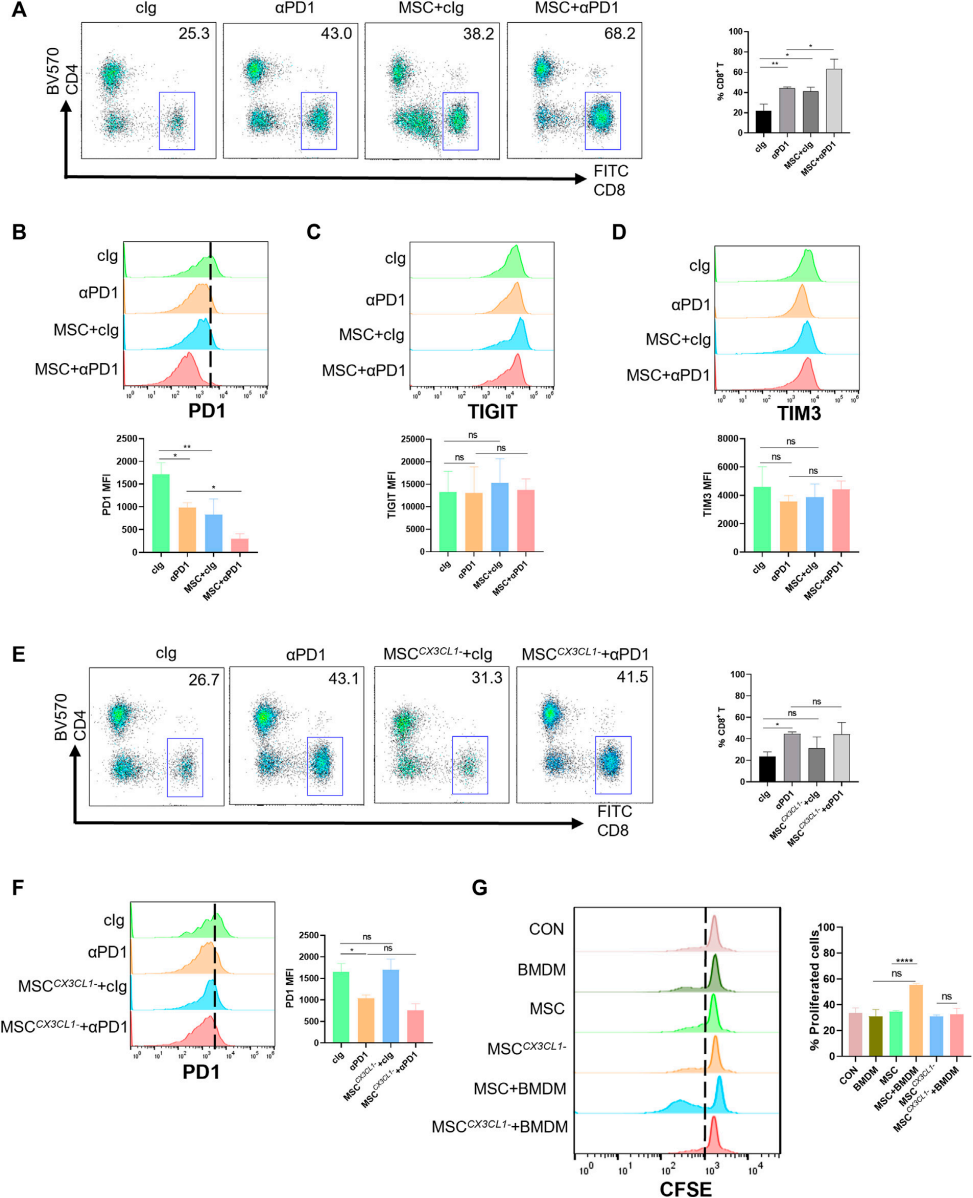
The promotion and modification effect of MSC for CD8^+^ T cells **(A)** Tumor-infiltrating CD8^+^ T cells were analyzed by FACS (gate on CD8^+^) (cIg-treated group, *n* = 3; αPD1-treated group, *n* = 3; MSC × cIg-treated group, *n* = 3; αPD1 × MSC mixed treatment group *n* = 3.) **(B–D)** Expression of PD1, TIGIT, and TIM3 in CD8^+^ T cells were analyzed by FACS (*n* = 3) **(E)** Tumor-infiltrating CD8^+^ T cells were analyzed by FACS (gate on CD8^+^) [cIg-treated group, *n* = 3; αPD1-treated group, *n* = 3; MSC ^*CX3CL1-*^-treated group, *n* = 3; αPD1 × MSC ^*CX3CL1-*^ mixed treatment group *n* = 3.] **(F)** Expression of PD1 in CD8^+^T were analyzed by FACS (*n* = 3) **(G)** CFSE-labeled mouse CD8^+^ T cells were treated with various groups. CD8^+^ T-cell proliferation was was analyzed by FACS (*n* = 3). Data are shown as means ± SD of the percentages of proliferated CD8^+^ T cells. Significance identification: ns, *p* ≥ 0.05; *, *p* < 0.05; **, *p* < 0.01; ***, *p* < 0.001. Mφ:macrophage.

CD8^+^ T cells are a significant component of adaptive immunity and play a major role in killing tumor cells (Yang et al., 2021). We analyzed the infiltration of CD8^+^ T cells into the TME (gating strategies in Supplementary Figure S1). The results showed that both αPD1 and MSC could improve the infiltration ratio of CD8^+^ T cells, and the αPD1 × MSC mixed group significantly increased the infiltration ratio of CD8^+^ T cells (Figure 6A). The immune checkpoint receptor (such as PD-1, TIGIT, and TIM3) is expressed in immune cells. We detected the expression of PD-1, TIGIT, and TIM3 in CD8^+^ T cells and found that MSC and αPD1 could inhibit the expression of PD1 in CD8^+^ T cells, and mixed treatment could significantly inhibit the expression of PD1 (Figure 6B). TIGIT and TIM3 levels did not change significantly in any of the experimental groups (Figures 6C, D). Inhibition of CX3CL1 secretion resulted in the disappearance of these effects (Figures 6E, F).

Macrophages play a connection role in specifically recognizing tumor antigens and targeting activated effector cells. Hence, we speculate that MSC function of increasing the proportion of CD8^+^ T cells infiltrating TME and reducing the expression of PD1 in CD8^+^ T cells may be presented by engineering macrophages. Based on these results, we designed a T-cell proliferation experiments. The results showed that CM from the MSC × BMDM co-culture groups significantly promoted the proliferation of CD8^+^ T cells, with no promotion in other groups (Figure 6G). Thus, MSC promoted the proliferation and reduced the expression of PD1 in CD8^+^ T cells by engineering macrophages, improved sensitivity to αPD1 treatment.

## 4 Discussion

Recent breakthroughs in cancer immunotherapies have dramatically strengthened the fight against cancer; blocking the PD1/PDL1 axis using monoclonal antibodies has been widely used in the clinical treatment of various tumors (Li et al., 2019). However, in many solid tumors, single αPD1 therapy fails to acquire ideal results. The inconsistency in response to blocking therapy prompted us to develop more specific and effective immunotherapies (El-Khoueiry et al., 2017). By utilizing the extensive chemokine expression profile of MSC, MSC-based immunotherapy actively inflamed tumors with immune effector cells, including macrophages and CD8^+^ T cells, and showed promising therapeutic effects in CRC (Rooney et al., 2015). Our results suggest that MSC can inhibit the growth of colorectal cancer and significantly increase colorectal cancer sensitivity to αPD1 therapy.

MSC can influence tissue metabolism and inflammation and play an important role in tumor metabolic immunity (Spallanzani, 2021). It has been documented that MSC has different or even diametrically opposite functions in different tumors, and the mechanisms of action vary. MSC can downregulate VEGF expression and reduce angiogenesis, thereby inhibiting the progression of breast cancer or prostate cancer (Lee et al., 2013; Alcayaga-Miranda et al., 2016), and extracellular vesicles from MSC have been reported to activate negative regulators of the cell cycle, leading to apoptosis or necrosis in hepatocellular carcinoma, ovarian cancer, and Kaposi’s sarcoma (Bruno et al., 2013). Additionally, MSCs can promote tumor progression in some conditions. MSC promotes proliferation, migration, and tumorigenesis in nasopharyngeal carcinoma and osteosarcoma (Shi et al., 2016; Zhao et al., 2019). MSC-derived extracellular vesicles have similar effects in renal, lung, and breast cancers (Du et al., 2014; Dong et al., 2018; Zhou et al., 2019). However, the function of MSC in CRC remains unclear. Our experiments in mice showed that MSC inhibited the growth and proliferation of CRC cells *in vitro* and *in vivo*, providing a new theoretical basis for clarifying the function of MSC.

Macrophages are a highly plastic group of cells, and different cell subsets can polarize in different directions upon TME stimulation (Davies et al., 2013). Macrophages in mice can be divided into two main subsets based on their surface molecule expression: F4/80^+^ CX3CR1^low^ cells or F4/80^+^ CX3CR1^high^ cells (Koscsó et al., 2020). In this study, we focus on the CX3CR1^high^ macrophage subset, which showed significant changes in CRC, whose function in the TME also remains undefined. This subset polarizes toward M2 macrophages in breast cancer and promotes tumor progression but polarizes toward M1 macrophages in melanoma and inhibits tumor growth (Franklin et al., 2014; Kubo et al., 2017). MSC exerts a significant regulatory effect on macrophages (Cho et al., 2014). It is feasible to modify the TME using MSC and change the polarization direction of macrophages to reverse the tumor immune microenvironment and effectively inhibit tumor progression. Following the above hypothesis, our experiments revealed that MSC recruited macrophages dominated by the CX3CR1^high^ subset and engineered them for M1 polarization in CRC, thereby inhibiting CRC growth and proliferation. Meanwhile, we found that MSC did not have a significant effect on macrophages M2 polarization. So we speculated that the therapeutic effect of MSC on CRC might mainly focus on M1 polarization. Our experiments indicated that the future direction of MSC-based immunotherapeutic strategy might also focus on promoting macrophages M1 polarization. Indirect co-culture experiments of MSC × macrophages suggests that MSC may act through extracellular vesicles or soluble secreted factors (cytokines or chemokines). The specific mechanisms in the combination of MSC and macrophages still needs to be further researched.

Macrophage-predominant innate immune cells can activate CD8^+^ T cells to specifically attack tumor cells and are considered one of the most effective immune responses (Yang et al., 2021). However, the immunosuppressive properties of the TME can inhibit CD8^+^ T cells activity, which may be responsible for the failure of antibody-blocking therapy (Sangro et al., 2021). Reversing the immunosuppressive characteristics of TME and promoting CD8^+^ T cells infiltration or activation by recruiting and engineering relevant macrophages can effectively improve the sensitivity of antibody-blocking therapy (Klug et al., 2013). In our experiments, CD8^+^ T cells were extensively exhausted in the TME, and PD1 was highly expressed in CD8^+^ T cells. Therefore, we evaluated the effect of MSC on the CRC TME. We found that MSC, in combination with αPD1 therapy, effectively inhibited the expression of PD1 in CD8^+^ T cells and promoted CD8^+^ T cells proliferation in tumors by engineering CX3CR1^high^ macrophages, improving sensitivity to anti-PD1 antibodies. But the detailed pathways involved in the combination of macrophage and CD8^+^ T cells warrant further investigation. Additionally, associated secreted proteins representing CD8^+^ T cell function (such as granzyme A/B、perforin、IL-2、IFN-r) need further reserch.

These above results might explain why the combination of MSC and αPD1 can better inhibit CRC growth in mice than αPD1 monotherapy. However, our murine model does not represent the heterogeneity of all CRC cases. The effect of MSC combined with αPD1 should be evaluated in different CRC models (Bürtin et al., 2020). Moreover, our study lacks clinical validation; therefore, the actual clinical therapeutic effect of MSC combined with anti-PD1 antibodies in patients with CRC needs further study.

**FIGURE 7:**
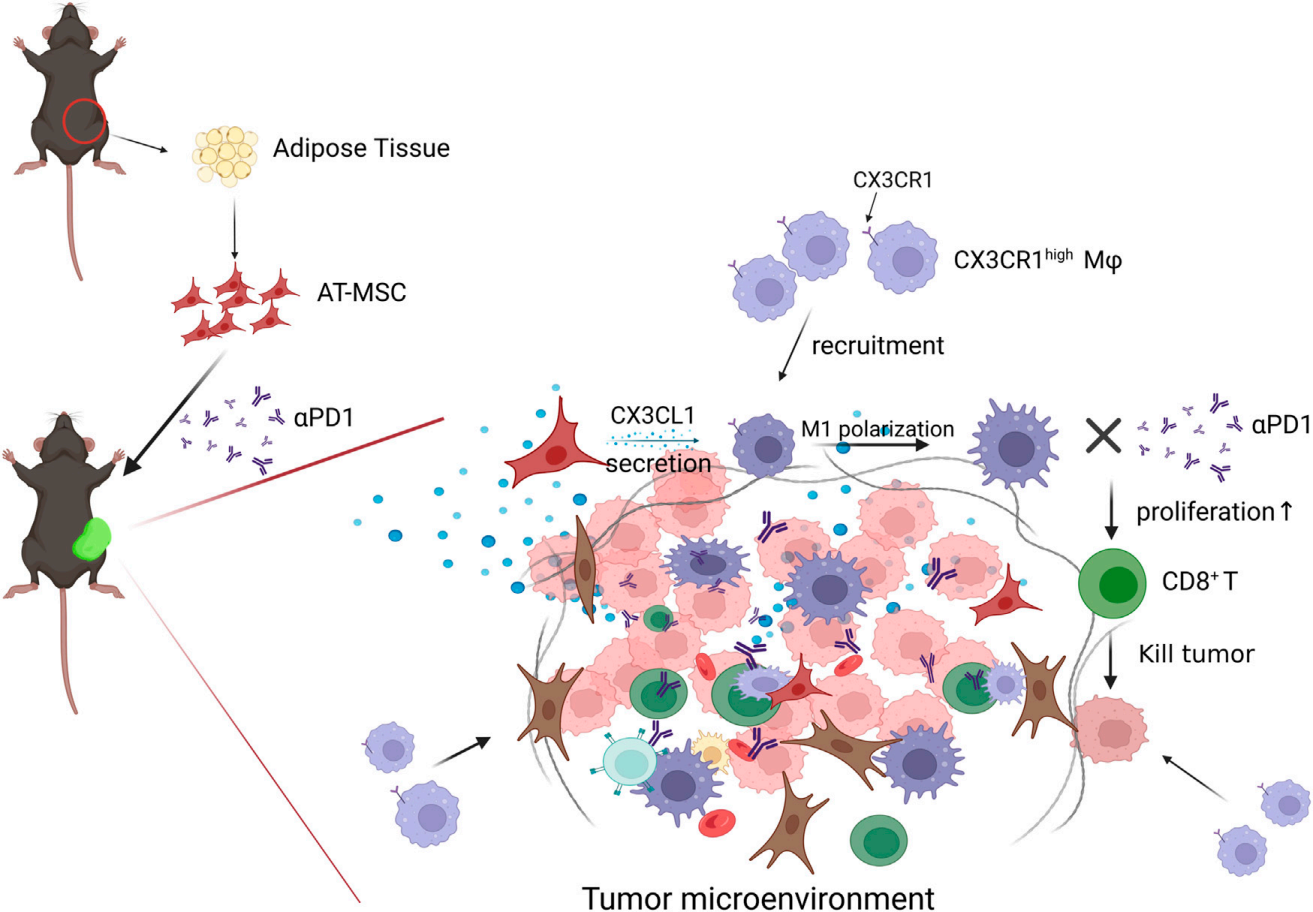
Diagram of possible mechanism of MSC MSC recruited CX3CR1^high^ macrophages and promoted their M1 polarization, which stimulated CD8^+^ T cells proliferation and activation, or inhibited PD1 expression on CD8^+^ T cells, ultimately ameliorating the immunosuppressive TME and attenuating CD8^+^ T cells tumor-killing activity in CRC.

In conclusion, our study revealed that MSCs inhibit CRC growth, and the combination of MSC and αPD1 could suppress CRC tumor progression in mice and was better than single therapy. MSC recruited more CX3CR1^high^ macrophages and promoted their M1 polarization, which stimulated CD8^+^ T cells proliferation and activation, or inhibited PD1 expression on CD8^+^ T cells, ultimately ameliorating the immunosuppressive TME in CRC (Figure 7). The combination of MSC and anti-PD1 antibodies may be a potential therapeutic strategy for CRC treatment.

## Data Availability

The original contributions presented in the study are included in the article/Supplementary Material; further inquiries can be directed to the corresponding authors.

## Abbreviations

BMDM; bone marrow-derived macrophages, CRC; colorectal cancer, DMEM; Dulbecco’s modified Eagle medium, MSC; mesenchymal stem cells, PD1; programmed cell death protein-1, TME; tumor microenvironment, αPD1; anti-PD1 antibodies.
