# Supplementary material for: Mesenchymal stem cells elicits Anti-PD1 immunotherapy by targeted delivery of CX3CL1
Source: Front Pharmacol. 2023 Feb 8;14:1136614. doi: 10.3389/fphar.2023.1136614 (PMC9944415; doi:10.3389/fphar.2023.1136614)
Supplement: Supplementary file 1 [file Table1.DOCX]

CD45 Biolegend Cat：103138

CD11b Biolegend Cat：101206

F4/80 Biolegend Cat：123133

CX3CR1 Biolegend Cat：149007

CD3 Biolegend Cat：100270

CD8 Biolegend Cat：100706

CD206 Biolegend Cat：141723

I^A^I^E^ Biolegend Cat：107664

CD29 Biolegend Cat：102215

CD44 BD Horizon Cat：563970

CD45 Biolegend Cat：103138

CD34 BD Horizon Cat: 551387

TIGIT Biolegend Cat：142111

TIM3 Biolegend Cat：134021

These antibodies were diluted according to the manufacturer’s instructions.

The primers were designed as follows:

5′- CATTGCTGACAGGATGCAGAAGG -3′ and 5′- TGCTGGAAGGTGGACAGTGAGG -3′ for beta-actin.

5′- ACGAAATGCGAAATCATGTGC -3′ and 5′- CTGTGTCGTCTCCAGGACAA -3′ for CX3CL1.
