# Supplementary figures and images for: Mesenchymal stem cells elicits Anti-PD1 immunotherapy by targeted delivery of CX3CL1
Source: Front Pharmacol. 2023 Feb 8;14:1136614. doi: 10.3389/fphar.2023.1136614 (PMC9944415; doi:10.3389/fphar.2023.1136614)

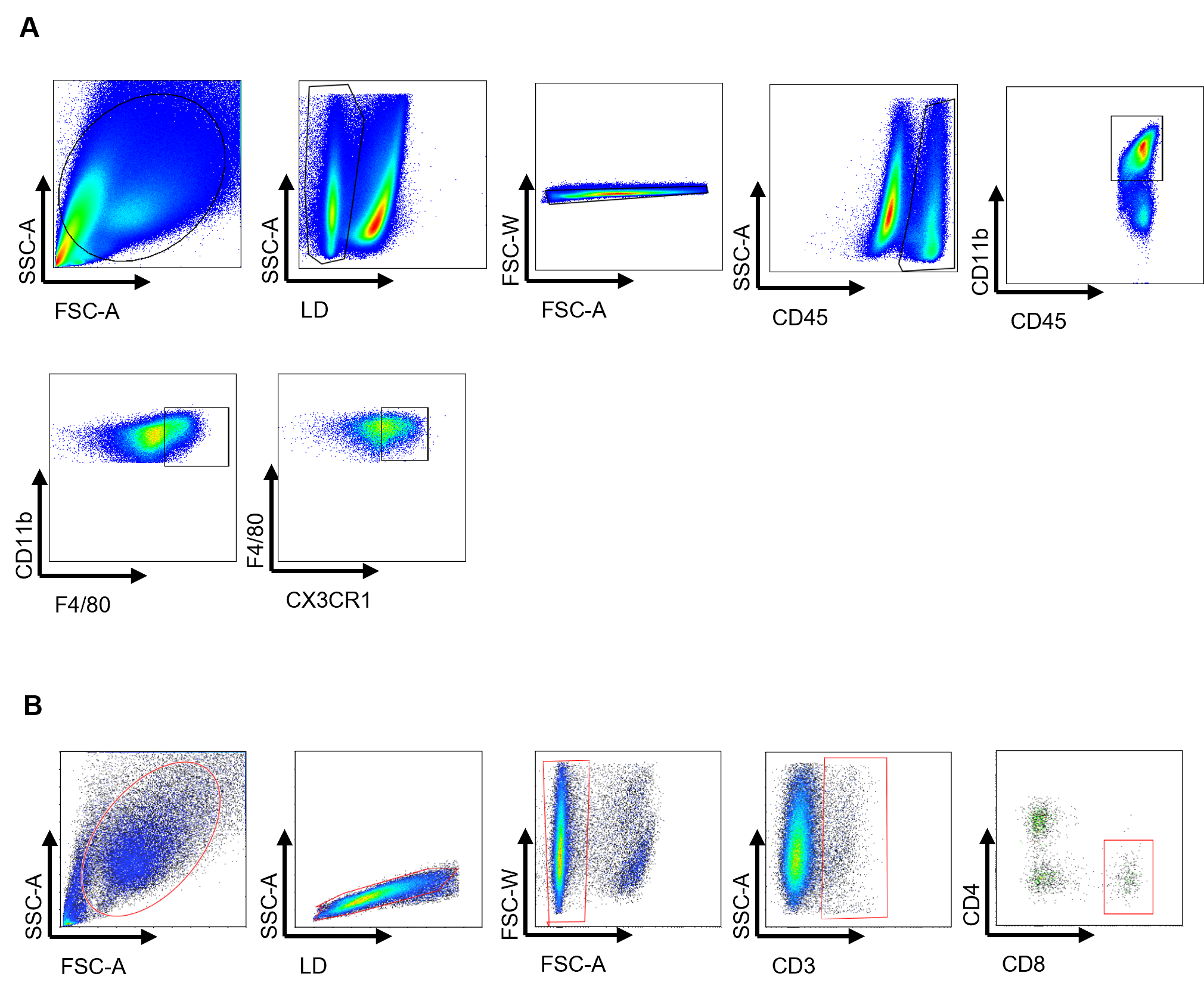

Supplement: Supplementary file 2 [file Image1.TIF]
